# Risk Factors for Osteoporosis among Patients with Inflammatory Bowel Disease—Do We Already Know Everything?

**DOI:** 10.3390/nu15051151

**Published:** 2023-02-24

**Authors:** Konrad Lewandowski, Magdalena Kaniewska, Martyna Więcek, Paulina Szwarc, Paulina Panufnik, Edyta Tulewicz-Marti, Magdalena Walicka, Edward Franek, Grażyna Rydzewska

**Affiliations:** 1 Clinical Department of Internal Medicine and Gastroenterology with Inflammatory Bowel Disease Unit, National Medical Institute of the Ministry of the Inferior and Administration, 02-507 Warsaw, Poland; 2 Department of Internal Medicine, Endocrinology and Diabetology, National Medical Institute of the Ministry of the Inferior and Administration, 02-507 Warsaw, Poland; 3 Mossakowski Medical Research Institute, Polish Academy of Science, 02-106 Warsaw, Poland; 4 Collegium Medicum, Jan Kochanowski University, 25-317 Kielce, Poland

**Keywords:** metabolic bone disease, inflammatory bowel diseases, osteoporosis, vitamin D, physical activity

## Abstract

Introduction: There are many known risk factors for osteoporosis (OST) among patients with inflammatory bowel disease (IBD), one of which is physical activity. Material and methods: The aim of the study is to assess the frequency and risk factors of OST among 232 patients with IBD compared to a group of 199 patients without IBD. The participants underwent dual-energy X-ray absorptiometry, laboratory tests, and completed a questionnaire about their physical activity. Results: It was found that 7.3% of IBD patients suffered from OST. Male gender, ulcerative colitis, extensive inflammation in the intestine, exacerbation of disease, rare physical activity, other forms of physical activity, past fractures, lower levels of osteocalcin, and higher levels of C-terminal telopeptide of type 1 collagen were risk factors for OST. As many as 70.6% of OST patients were rarely physically active. Conclusions: OST is a common problem in IBD patients. OST risk factors differ significantly between the general population and those with IBD. Modifiable factors can be influenced by patients and by physicians. The key to OST prophylaxis may be regular physical activity, which should be recommended in clinical remission. It may also prove valuable to use markers of bone turnover in diagnostics, which may enable decisions regarding therapy.

## 1. Introduction

Inflammatory bowel disease (IBD) includes ulcerative colitis (UC) and Crohn’s disease (CD) [1,2]. In the course of this disease, apart from the involvement of the gastrointestinal tract, up to 50% of patients may also have extraintestinal manifestations (EIMs) [3]. One of them may be osteoporosis (OST), which is present in over half of IBD patients; this constitutes a significant problem due to the increased risk of fractures, especially non-traumatic fractures [3].

Known risk factors for OST in IBD include the persistence of chronic inflammation, treatment with glucocorticosteroids, extensive range of the disease, previous resection of the small intestine, arthralgia, and current nutritional deficiencies. Some risk factors are similar to those found in the general population, such as age, smoking, or low physical activity [3]. The gold standard in the diagnosis of OST is dual-energy X-ray absorptiometry (DXA) performed on the femoral neck and the lumbar spine. The World Health Organization (WHO) defined a range for DXA reference values in relation to the T-score, but these values were based on recommendations for postmenopausal Caucasian women and there are insufficient data to extrapolate them to other patient groups [3,4].

Among modifiable osteoporosis risk factors specific to IBD, appropriate disease control seems to be a crucial one, especially taking into account the treat-to-target model of treatment [5]. There is some evidence that bone density may normalize after 3 years of stable remission of IBD [6]; nevertheless, even in those patients, other risk factors remain and action should be taken to mitigate them. Actions that can be beneficial in preventing bone mineral density (BMD) loss are doing weight-bearing exercises, quitting smoking, and maintaining an adequate amount of calcium in the diet [1000 mg/day]. The beneficial role of calcium and vitamin D supplementation in patients taking systemic glucocorticosteroids has also been demonstrated throughout the therapy [5].

We have irrefutable evidence of the beneficial effects of physical activity for many disease entities. Physical exercise also affects the molecular pathways of bone remodeling, involving all types of bone tissue cells. Unfortunately, we do not have similar data in patients with IBD, although OST is commonly observed in this population. The type and frequency of physical activity, as a modifiable risk factor for OST, needs to be specified in order to make appropriate recommendations for patients. In the general population, multi-component training seems to be the most promising, including aerobic activity and other types of training (resistance and/or strength exercises), because it improves BMD–especially in the elderly and in post-menopausal women [5].

All this indicates the presence of many factors that predispose patients to the development of OST, as well as several factors that have a protective effect on prevention. Nevertheless, some of them still do not have a clearly defined role, which makes the research results inconsistent. The role of physical activity as a protective factor is obvious, though its individual types remain unexplored. In the analysis below, we have included jogging, cycling, gymnastics, swimming or other activities, taking into account the frequency, which seems to be of key importance. In addition, due to the limited number of studies on bone turnover parameters in IBD patients, we examined the values of C-terminal telopeptide of type 1 collagen (CTX), osteocalcin, parathyroid hormone (PTH), and vitamin D.

## 2. Materials and Methods

The aim of the study is to assess the frequency and risk factors of OST among patients with IBD compared to a control group without IBD. We conducted a single-center analysis of the frequency of osteoporosis in patients aged 18 and older who had confirmed IBD and in a control group without IBD at the Central Clinical Hospital of the Ministry of the Interior and Administration in Warsaw, Poland. All patients in both study groups raised suspicions of OST and were therefore subjected to DXA (2014 Prodigy PRO model) and blood tests to assess bone metabolism with C-terminal telopeptide of CTX, osteocalcin, PTH, and vitamin D levels, and were given an OST risk factor questionnaire. The OST diagnosis was based on the DXA score, which was measured at the femoral neck and lumbar–sacral spine for the following values if the T-score equaled or exceeded −2.5 SD. The study protocol was approved by the Bioethics Committee of the Central Clinical Hospital of the Ministry of the Interior and Administration in Warsaw. The study lasted two years, from December 2019 to December 2021.

### Statistical Analysis

Statistical analyses were run for patients with IBD (*n* = 232) and the control group (*n* = 199) separately, and the outcomes were compared. The group characteristics are presented for each study group. Categorical variables are described with the number of observations and the percentage frequency. Numerical variables are described with means and standard deviation or medians and 1st and 3rd quartiles, depending on normality. Normality was tested with the Shapiro–Wilk test and verified with skewness and kurtosis. Homogeneity was tested with Levene’s test. For continuous parameters, differences between groups were assessed with Student’s independent t-test (in the case of normal distribution and homogeneity of variance), Welch’s independent t-test (in the case of normal distribution and no homogeneity of variance), or Mann–Whitney U test (in the case of non-normal distribution). Differences between groups for categorical parameters were assessed with Pearson’s Chi-square test or Fisher’s exact test, depending on whether the assumption for expected counts for Pearson’s Chi-square test was satisfied, and described with Cramer’s V. A logistic 2-step regression approach was used to identify significant predictors of osteoporosis and osteopenia risk (univariate models for each variable in the first step and multivariate models as the second step). Variable selection for multivariate models was based on the p-value produced in the first step being no higher than 0.250. Multivariate models were assessed with Nagelkerke’s R^2^ and Hosmer–Lemeshow tests. The predictors’ effect is described with an odds ratio (OR) along with 95% confidence intervals. All statistical calculations assumed an *alpha* value of 0.05. Analysis was run in R software, version R-4.1.2.

## 3. Results

### 3.1. Characteristics of the Groups

The group with IBD consisted of 232 patients, 52.2% of whom were women. The average age was 42.09 ± 14.38 years. The control group without IBD consisted of 199 patients, 70.4% of whom were women. The average age was 54.00 ± 12.57 years. The subjects in the two groups demonstrated demographic differences. The patients with IBD were almost 12 years younger (*MD* = −11.91, CI_95_ [−14.49;−9.33], *p* < 0.001), and while there was a roughly equal proportion of men and women within the IBD group (52.2% vs. 47.8%, respectively), female sex prevailed in the control group (70.4%). The level of correlation between sex and IBD was low, based on Cramer’s V (*V* = 0.19, CI_95_ [0.09;0.27], *p* < 0.001).

Additionally, the proportion of smokers was lower among patients with IBD (11.6% vs. 21.1% in the control group), with a weak association measured by Cramer’s V (*V* = 0.13, CI_95_ [0.04;0.22], *p* = 0.011). The frequency of physical activity differed between the groups. The correlation between having IBD and the frequency of physical activity was at a moderate level (*V* = 0.39, CI_95_ [0.32;0.46], *p* < 0.001). The main difference lay in patients with daily physical activity (28.0% of the patients with IBD, 3.5% (*n* = 7) of the control group). There were also more patients exercising once a week in the IBD group (32.8% vs. 23.6% in the control group), while fewer people declared rarely doing physical activity (24.6% vs. 47.7% in the control group). No physical activity was noted for 14.7% of the IBD group and for 25.1% of the control group. In addition, an analysis comparing the performance of resistance exercise (gymnastics vs. physical activities other than exercise) was conducted, finding that 80.1% of IBD patients performed non-resistance exercise (*p* < 0.001). Supplementation of vitamin D and calcium was significantly more common among patients suffering from IBD (31.0% vs. 17.5% in the control group) with a low strength of association (*V* = 0.16, CI_95_ [0.06;0.24], *p* = 0.002). Fracture history appeared less often among patients with IBD than in the control group (7.8% vs. 19.6%). The association was measured by Cramer’s V was weak (*V* = 0.17, CI_95_ [0.09;0.26], *p* = 0.001). Arthralgia was noted more often within the IBD group than the control group (50.9% vs. 38.2%) and the association between arthralgia and IBD had low strength (*V* = 0.13, CI_95_ [0.03;0.22], *p* = 0.011). The other parameters did not vary between the groups significantly (*p* > 0.05).

Among patients with IBD, osteoporosis was diagnosed in 17 cases (7.3%). Moreover, osteopenia was found in one-third of patients (*n* = 78, 33.6%). These values did not differ significantly from the frequency of osteoporosis (*n* = 15, 7.5%) or osteopenia (*n* = 69, 34.7%) in the control group (*MD* = 0.01, CI_95_ [0.01;0.14], *p* = 0.956). All data are presented in Table 1.

### 3.2. Risk Factors for Osteoporosis in Patients with IBD

**Figure 1:**
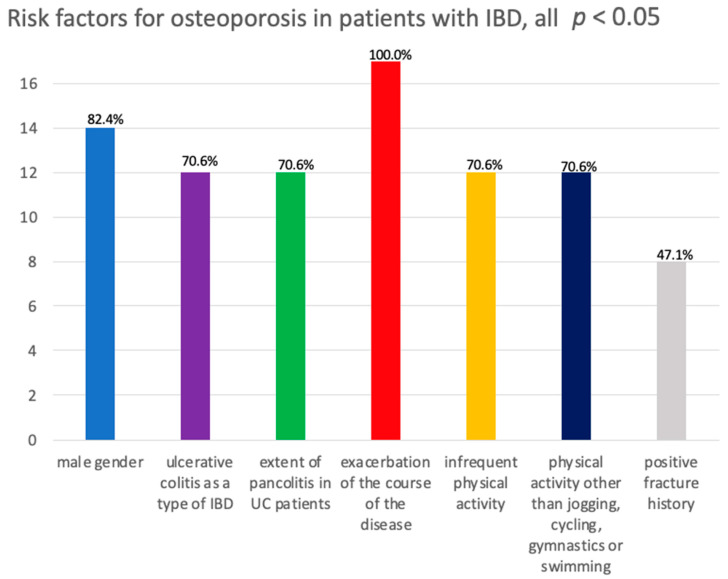
Risk factors for osteoporosis in patients with IBD.

The risk factors for osteoporosis were male gender, ulcerative colitis as the type of IBD, an extensive range of inflammation in the intestine (pancolitis) among UC patients, exacerbated course of the disease, infrequent physical activity, physical activity other than jogging, cycling, gymnastics or swimming, positive fracture history and abnormalities of certain bone turnover markers: a higher level of C-terminal telopeptide of CTX and a lower level of osteocalcin. All variables had a significant effect, but most were weak (Cramer’s V, 0.1–0.3; Figure 1).

The male gender was more frequent within the osteoporosis group (82.4%; *V* = 0.19, CI_95_ [0.08;0.30], *p* = 0.007). Contrary to previous reports, patients with UC (70.6%) were more likely to suffer from OST, whereas CD was less common (29.4%; *V* = 0.22, CI_95_ [0.08;0.34], *p* = 0.002). The incidence of pancolitis, as an extensive localization of UC, significantly differed between OST and non-OST groups (70.6% vs. 19.5%, respectively; *V* = 0.31, CI_95_ [0.16;0.46], *p* < 0.001). Unsurprisingly, exacerbation was observed in all patients with osteoporosis, making disease remission the ultimate goal of OST prevention. Frequency of physical activity differentiated the two subgroups significantly (*V* = 0.33, CI_95_ [0.22;0.45], *p* < 0.001) because the majority of patients with OST were rarely physically active (70.6%). A well-known risk factor—fracture history—was obviously more frequent among patients with OST (47.1% vs. 4.7%, respectively; *V* = 0.44, CI_95_ [0.18;0.64], *p* < 0.001). Interestingly, there was a lower average osteocalcin value in the OST group (*MD* = −9.30, CI_95_ [−12.10;−1.20], *p* = 0.007), which was unsatisfactory because we hypothesized higher values of the bone formation marker here. However, these discussions were confirmed in CTX (CTX: *MD* = 0.06, CI_95_ [0.01;0.22], *p* = 0.045). For the other parameters under analysis, no significant difference between the groups was confirmed (*p* > 0.05; Table 2).

### 3.3. Logistic Regression Model for Osteoporosis in Patients with IBD

Two-step logistic regression was run to identify the risk factors for OST among the patients with IBD. The first step consisted of univariate logistic regression models for each predictor variable. Statistical significance levels were the main criterion for selecting variables for the second step, the multivariate logistic regression model. It was assumed that the p-value from the univariate models must satisfy the condition of *p* < 0.250. The fit of the multivariate model and the collinearity of the independent variables were also considered in the final selection of predictors. All data are presented in Table 3.

For patients with IBD, male gender was associated with an almost sixfold higher risk of osteoporosis (*OR* = 5.68, CI_95_ [1.79;25.14], *p* = 0.008). Crohn’s disease, compared to UC, lowered the osteoporosis risk by 81% (*OR* = 0.19, CI_95_ [0.06;0.53], *p* = 0.003). UC within pancolitis was associated with a tenfold higher risk (*OR* = 9.89, CI_95_ [3.47;32.50], *p* < 0.001). Rare physical activity brings a threefold higher risk (vs daily activity; *OR* = 3.20, CI_95_ [1.10;10.66], *p* = 0.040). Patients with physical activity other than jogging, cycling, gymnastics, or swimming had a fivefold higher risk of osteoporosis (*OR* = 5.42, CI_95_ [1.93;17.60], *p* = 0.002). Fracture history increases the risk 18 times (*OR* = 18.22, CI_95_ [5.79;58.70], *p* < 0.001). Additionally, a range of numeric parameters influenced the risk level. An osteocalcin level that is one unit higher would cause a 6% lower osteoporosis risk (*OR* = 0.94, CI_95_ [0.88;0.98], *p* = 0.018). Statistical significance was not confirmed for other predictor variables (*p* > 0.05).

The multivariate regression step identified variables that influence the risk of osteoporosis when working in combination. Significant influences were confirmed for age, gender, Crohn’s disease (vs. UC), rare physical activity (vs. daily), and other physical activity types. Age lowered osteoporosis risk by 24% (*OR* = 0.76, CI_95_ [0.56;0.92], *p* = 0.021). Male gender was associated with an almost 6000 times higher risk of osteoporosis (*OR* = 5937.15, CI_95_ [19.70;4.40 × 10^8^], *p* = 0.028). Crohn’s disease, compared to UC, lowered the osteoporosis risk (*OR* = 0.00, CI_95_ [0.00;0.05], *p* = 0.007). Rare physical activity brought an almost 380 times higher risk (vs. daily; *OR* = 374.85, CI_95_ [7.74;339510.40], *p* = 0.023). Performing other physical activity was associated with an almost 140 times higher risk of developing osteoporosis (OR = 138.50, CI_95_ [3.03;34011.20], *p* = 0.033). The other predictors analyzed in the multivariate regression did not have a significant impact on risk. The fit of the multivariate model was assessed with Nagelkerke’s R^2^, which was found to be 83.6%, indicating a very good fit of the model. The Hosmer–Lemeshow test resulted in a value of *p* = 0.993, confirming a good model fit.

### 3.4. Comparison between Patients with IBD and Patients from the General Population

In terms of diagnosed osteoporosis, a significant correlation was found between patients with IBD and the general population for age, sex, physical activity, and arthralgia. The IBD patients with osteoporosis were more than 7 years younger than the general population (39.29 vs. 46.87; MD −7.57; *p* = 0.033). In men with IBD, osteoporosis was found much more often (82.4% vs. 33.3%; *V* = 0.50; *p* = 0.014). Physical activity was more often performed by patients with IBD (*V* = 0.53; *p* = 0.015), and among this group, arthralgia was more frequent (52.9% vs. 13.3%; *V* = 0.42; *p* = 0.048; Table 4).

## 4. Discussion

Worryingly, the data show an increase of more than 28% in the annual number of osteoporotic fractures, which will increase from 3.5 million in 2010 to an estimated 4.5 million in 2025 [7]. In addition, there are data in the USA estimating the cost of healthcare associated with fractures to be $12–18 billion [8]. The risk factors for OST in the general population seem to be well defined and it seems realistic to identify those patients who require supervision in this area, thanks to well-organized healthcare [4]. Unfortunately, these recommendations cannot be extrapolated to the IBD patient population in Poland, which is currently estimated at 23,574 patients with CD and 73,235 with UC [9]. Similarly to the authors of other studies, we considered the relationship between OST and IBD and the risk factors for OST, where some are known (age, female gender, and low BMI) and others still diverge. However, the preventive role of physical activity has not been fully defined; in particular, its type and frequency remain a mystery.

In a 2020 systematic review of the prevalence and development of OST or low bone mineral density and its risk factors in patients with IBD, Kärnsund et al. identified the following: CD diagnosis, lower BMI, and lower body weight [5]. In 2014, in Switzerland, Schüle et al. reviewed 877 patients with IBD to evaluate the details of the diagnostics and treatment of osteoporosis. It was found that osteoporosis occurred in 20% of the patients studied, while the use of glucocorticosteroids, long duration of the disease, and perianal disease were considered risk factors for the development of OST. In addition, suboptimal rates of treatment for OST patients were described (55% for calcium and 65% for vitamin D) [10]. Ratajczak et al. reported similar results for vitamin D concentration [11].

Our study showed how common the problem of OST (7.3%) and more than one-third of osteopenia (33.6%) in IBD patients can be. There have been some interesting observations regarding risk factors: male gender, ulcerative colitis (UC), location of pancolitis in UC, exacerbation of the disease, infrequent physical activity, other forms of physical activity, history of fractures, lower levels of osteocalcin and higher levels of the C-terminal telopeptide of CTX. Moreover, it is worrying that the average level of vitamin D3 was 16.20 ng/mL, which is far below normal.

Ratajczak et al. presented some disturbing results—five times higher than ours, where OST was present in almost 36% of subjects. On the other hand, there were fewer patients with osteopenia (about 24%) [11]. Our results are in line with the study by Kärnsund et al., where the frequency of osteoporosis was estimated at 2–15% and among healthy controls at 3–10% [5]. In our study, the incidence of OST was similar for both populations, but IBD patients were about 12 years younger on average. Patients with IBD more often supplemented vitamin D and calcium than members of the control group (31.0% vs. 17.6%; *V* = 0.16; *p* = 0.002). This can be explained by the fact that in our center we strictly follow the recommendations to include vitamin D and calcium supplementation. Unfortunately, as shown by the results of the average vitamin D concentration, it is too low, which was also confirmed in the results of Ratajczak [11]. Similarly, Leslie et al. found that only 21.8% of IBD patients had optimal vitamin D levels, indicating the need to raise the dosage in patients already using it and to initiate it in patients with risk factors [12]. This can be explained by the low compliance of our patients, which could have contributed to such a result.

Most often, osteoporosis occurs in female patients with IBD; however, it should be mentioned that some data suggest it is also a protective factor [13]. The data presented by Johansen et al. confirm our finding that OST may be more common in men [14]. Our study is the first to show an association between UC diagnosis (70.6%) and the location of pancolitis (70.6%) as a risk factor for the development of OST. In our study, all patients with OST had an exacerbated disease, which is the main risk factor for low bone mass in IBD, as described by Turk et al. [15]. Reports on the protective effect of physical activity on OST emphasize the need for moderate to frequent exercise. In our study, 70.6% of OST patients rarely engaged in physical activity, which, according to the results of studies by Mauoro et al. and Lee et al., predisposes the individual to low bone mass [16,17,18]. It was not surprising that almost half of the patients had had fractures, which was also found in two studies by Vestergaard et al., who found they were ×5 and ×7more likely to have any fractures among IBD [19,20].

Moreover, lower levels of osteocalcin and higher levels of C-terminal telopeptide of CTX were confirmed as risk factors for OST. In the group of patients with OST, it was found that the mean level of osteocalcin was lower (12.62 ng/mL vs. 22.00 ng/mL, *p* = 0007), which is a mystery. However, high levels of osteocalcin are usually due to the accelerated bone metabolism and extensive bone remodeling that is present in OST patients, with a few exceptions. The condition for a higher concentration of osteocalcin is the correct level of vitamins D and K2, which correspond to a regulation of its production [21]. In our study, this theory is partially supported by the low vitamin D level (14.60 ng/mL). Unfortunately, we did not test the level of vitamin K2. Additionally, the values of osteocalcin, which should raise concerns about the occurrence of OST, remain unclear [22,23,24,25,26,27,28]. CTX is a marker of bone resorption and it was significantly higher in the patient group with IBD and OST (0.49 mg/mL vs. 0.43 mg/mL, *p* = 0.045). Both of these markers may be elevated in osteoporosis, osteopenia and other endocrine disorders (hyperthyroidism). Apart from the work of Tulewicz et al., not much research has been carried out on bone mineral changes in IBD patients [29].

In our multivariable logistic regression model, male sex (*p* = 0.022) and physical activity of rare frequency (vs daily; *p* = 0.007) were associated with OST development. In the group of patients with OST, female gender was found to be a risk factor, which mainly concerns women in menopause and is associated with estrogen deficiency. However, the results of our study clearly highlight significant differences in terms of OST risk factors between patients with IBD and the general population. In our study, OST was found in 14 men with IBD, all of whom had an exacerbation at the same time, which may have contributed to this result, as an exacerbation of IBD can lead to malabsorption, which may be exacerbated by persistent inflammation [3,5]. Moreover, CD vs. UC reduced the risk of osteoporosis by 97% (*OR* = 0.03, CI95 [0.00; 0.36], *p* = 0.016). Another type of physical activity was not related to OST in this model, which we suspect could be related to the presence of a weak association (*V* = 0.22). An interesting relationship concerns past fractures, where the association was moderate (*V* = 0.41), but it may be an indicator of an unknown variable related with OST. The evaluation of our model shows that it was very well-fitted (Nagelkerke’s R2 = 77.3%, Hosmer–Lemeshow *p* = 0.999), which indicates the absence of other unassessed variables.

We also checked whether the factors responsible for OST among IBD patients differed from those presented in the general population. No statistical differences were found in terms of BMI, smoking, calcium in the diet, vitamin D and calcium supplementation, past fractures, and selected indicators of bone turnover. The most striking difference was seen in the case of rare physical activity (*V* = 0.53; *p* = 0.015). Other factors included age, gender, and arthralgia.

Physical activity is a factor that may improve BMD in the IBD population as well [30]. In 2021, Sigurdsson et al. examined the relationship between BMD and exercise habits in patients with childhood-onset IBD. They reported that 57% of them practiced physical activity, defined as > 4 h/week. In contrast, the sedentary patients had significantly lower median BMD (*p* < 0.05). The BMD results of patients with active IBD were comparable to those of the control group (*p* = 0.151). In the conclusions of the study, it was emphasized that frequent physical activity prevents the long-term effects of the disease [31]. In our study, we did not analyze the duration of the disease, which should be noted as a limitation. However, they analyzed different types of physical activity, which remains important given the potential need for specific recommendations for IBD patients. Rychter et al. showed that patients with CD who were diagnosed with osteopenia and OST performed less physical activity compared to patients with normal BMD (*p* = 0.0335) [16]. In our study, a similar relationship was found in the entire group of patients with IBD.

The analysis did not confirm a relationship between glucocorticosteroids used in IBD and the presence of OST. In the general population, this relationship has been unequivocally confirmed, while among IBD patients there are still too few studies, so the results are extrapolated here. It is known that the results of bone histology and BMD in patients treated with glucocorticosteroids vary depending on the dosage and duration of treatment. At the beginning of treatment, when higher dosages are usually used, severe resorption predominates; in chronic treatment, the reduction of bone formation predominates. When assessing their impact, one should take into account the fact that glucocorticosteroids are used in inflammatory diseases, which themselves cause osteoporosis, the severity of which depends on their activity. Undoubtedly, they also reduce the absorption of calcium in the intestines and increase its excretion in the urine, but this too was not observed in our results [5]. The absence of glucocorticosteroid effects on OST development in our study group may be related to the previously mentioned strict strategy of including calcium and vitamin D supplementation.

Similarly, no association with anti-TNFs was demonstrated. Data on the effects of these drugs on OST indicate a protective effect, though it is not known whether this is due to the mucosal healing effect in IBD or the effect against the TNF cytokine itself. Elevated TNF levels may play a role in disturbed bone metabolism in IBD. This cytokine induces osteoclast differentiation, increases bone resorption by osteoclasts, protects these cells from apoptosis, and reduces bone formation. Currently, five studies are available in which the protective effect of anti-TNFs was confirmed in small groups; however, as the authors of these studies themselves emphasize, the duration of treatment is crucial, mainly due to the fact that effective treatment enables the healing of inflammatory lesions [5,6]. In our study, OST was present in all patients who had an exacerbation of the disease, which in some way may emphasize the role of TNF as a cytokine that is detrimental to BMD.

### Strengths and Limitations

Some limitations of our study should be acknowledged: this is a single-center, observational study and most of the IBD patients were suffering exacerbations, which is an independent risk factor for OST. One strength of the study is the comparison of a group of patients with IBD with a control group, where the risk factors differed significantly. Moreover, to our knowledge, this is the first report to study the importance of not only frequent physical activity, but also the type of activity. Other strengths include the rare analysis of bone turnover markers and the good model fit, which is confirmed by the values of Nagelkerke’s R2 (77.3%) and the Hosmer–Lemeshow test result (*p* = 0.999).

## 5. Conclusions

Osteoporosis and osteopenia are common conditions in the IBD patient population. The risk factors for OST vary significantly between IBD patients and the general population. For this reason, IBD patients require a complete and more specialized approach. This may be related primarily to significant differences in risk factors, especially based on age or exacerbation of the disease. Among the risk factors for OST in patients with IBD, a distinction should be made between non-modifiable risk factors, those modifiable by patients (e.g., adhering to medical recommendations or doing physical activity), and those that can be influenced by the physician (e.g., selecting optimal treatment of exacerbations and making strict recommendations regarding vitamin D and calcium supplementation). Regular physical activity may be the key to OST prophylaxis, although it should be recommended for appropriate patients (e.g., not ill patients, due to exacerbations). It also seems interesting to use markers of bone turnover in the diagnosis, which may help in deciding on a therapy and may play an auxiliary role in determining the improvement of BMD. It is also crucial to expand the literature with multicenter studies to develop appropriate guidelines for monitoring IBD patients for metabolic bone disease.

## Figures and Tables

**Table 1:** Characteristics and comparison of study groups.

Variable	Patients with IBD	Control Group	MD/Cramer V (95% CI)	*p*
*N*	232 (100.0)	199 (100.0)	-	-
Age, years	42.09 ± 14.38	54.00 ± 12.57	−11.91 (−14.49;−9.33)	<0.001
Gender				
Female	121 (52.2)	140 (70.4)	0.19 (0.09;0.27)	<0.001
Male	111 (47.8)	59 (29.6)		
BMI	23.21 (20.06;25.80)	23.24 (19.88;26.37)	−0.03 (−0.78;0.76)	0.997
IBD				
Ulcerative colitis	79 (34.1)	-	-	-
Crohn’s disease	153 (65.9)	-	-	-
Crohn’s disease - location				
Small intestine	69 (29.7)	-	-	-
Large intestine	59 (25.4)	-	-	-
Ileocecal	73 (31.5)	-	-	-
Ulcerative colitis - location				
Pancolitis	54 (23.3)	-	-	-
Left-sided	5 (2.2)	-	-	-
Proctitis	20 (8.6)	-	-	-
Remission	87 (37.5)	-	-	-
Exacerbation	145 (62.5)	-	-	-
Smoking	27 (11.6)	42 (21.1)	0.13 (0.04;0.22)	0.011
Physical activity				
Everyday	65 (28.0)	7 (3.5)	0.39 (0.32;0.46)	<0.001
Once a week	76 (32.8)	47 (23.6)		
Rarely	57 (24.6)	95 (47.7)		
Not physically active	34 (14.7)	50 (25.1)		
Physical activity form				
Jogging	34 (14.7)	-	-	-
Cycling	51 (22.0)	-	-	-
Gymnastics	46 (19.8)	-	-	-
Swimming	38 (16.4)	-	-	-
Other	78 (33.6)	-	-	-
No information	33 (14.2)	-	-	-
Calcium in diet	49 (21.1)	30 (15.1)	0.08 (0.01;0.17)	0.136
Supplementation of vit. D and Ca	72 (31.0)	35 (17.6)	0.16 (0.06;0.24)	0.002
Glucocorticosteroids (latest 12 months)	90 (38.8)	-	-	-
Anti-TNF (latest 12 months)	36 (15.5)	-	-	-
Operation (latest 12 months)	20 (8.6)	-	-	-
Fracture	18 (7.8)	39 (19.6)	0.17 (0.09;0.26)	0.001
Arthralgia	118 (50.9)	76 (38.2)	0.13 (0.03;0.22)	0.011
Metabolic bone disease				
None	137 (59.1)	115 (57.8)	0.01 (0.01;0.14)	0.956
Osteopenia	78 (33.6)	69 (34.7)		
Osteoporosis	17 (7.3)	15 (7.5)		
CTX, ng/mL	0.52 ± 0.30	0.53 ± 0.30	−0.01 (−0.06;0.05)	0.780
Osteocalcin, ng/mL	20.40 (13.28;30.65)	20.40 (13.40;30.80)	0.00 (−2.30;1.90)	0.875
PTH, pg/mL	36.20 (26.75;47.60)	36.20 (26.00;47.60)	0.00 (−2.60;2.90)	0.969
Vit. D, ng/mL	16.20 (8.90;20.70)	16.20 (8.90;20.70)	0.00 (−1.50;1.40)	0.919

Data are presented as n (% of group), mean ± standard deviation or median (1st quartile; 3rd quartile), depending on the type of variable and the normality of distribution in the case of numeric variables. MD—mean or median difference (patients with IBD vs. patients without IBD), CI—confidence interval. Groups compared with Pearson’s Chi-square test or Fisher’s exact test (nominal variables) and Student’s t-test for independent groups or Mann–Whitney U test (numeric variables).

**Table 2:** Risk factors for osteoporosis in patients with IBD.

Variable	Risk Factors for Osteoporosis			
	Osteoporosis	No Osteoporosis	MD/Cramer V (95% CI)	*p*
*N*	17 (100.0)	215 (100.0)	-	-
Age, years	39.29 ± 5.03	42.31 ± 14.85	−3.02 (−6.21;0.18)	0.064
Gender				
Female	3 (17.6)	118 (54.9)	0.19 (0.08;0.30)	0.007
Male	14 (82.4)	97 (45.1)		
BMI	23.60 (15.51;23.60)	23.18 (20.06;25.80)	0.42 (−4.03;1.85)	0.462
IBD				
Ulcerative colitis	12 (70.6)	67 (31.2)	0.22 (0.08;0.34)	0.002
Crohn’s disease	5 (29.4)	148 (68.8)		
Crohn’s disease-location				
Small intestine	5 (29.4)	64 (29.8)	0.00 (0.00;0.14)	>0.999
Large intestine	5 (29.4)	54 (25.1)	0.03 (0.00;0.16)	0.773
Ileocecal	0 (0.0)	73 (34.0)	0.19 (0.14;0.24)	0.009
Ulcerative colitis-location				
Pancolitis	12 (70.6)	42 (19.5)	0.31 (0.16;0.46)	<0.001
Left-sided	0 (0.0)	5 (2.3)	0.04	>0.999
Proctitis	0 (0.0)	20 (9.3)	0.09 (0.06;0.11)	0.374
Remission	0 (0.0)	87 (40.5)	0.22 (0.16;0.28)	0.002
Exacerbation	17 (100.0)	128 (59.5)	0.22 (0.16;0.27)	0.002
Smoking	0 (0.0)	27 (12.6)	0.10 (0.07;0.13)	0.232
Physical activity				
Everyday	5 (29.4)	60 (27.9)	0.33 (0.22;0.45)	<0.001
Once a week	0 (0.0)	76 (35.3)		
Rarely	12 (70.6)	45 (20.9)		
Not physically active	0 (0.0)	34 (15.8)		
Physical activity form				
Jogging	0 (0.0)	34 (15.8)	0.12 (0.08;0.15)	0.144
Cycling	0 (0.0)	51 (23.7)	0.15 (0.11;0.19)	0.028
Gymnastics	5 (29.4)	41 (19.1)	0.07 (0.00;0.22)	0.342
Swimming	0 (0.0)	38 (17.7)	0.12 (0.09;0.16)	0.083
Other	12 (70.6)	66 (30.7)	0.22 (0.07;0.34)	0.002
No information	0 (0.0)	33 (15.3)	0.11 (0.08;0.15)	0.141
Calcium in diet	5 (29.4)	44 (20.5)	0.06 (0.00;0.19)	0.366
Supplementation of vit. D and Ca	5 (29.4)	67 (31.2)	0.01 (0.00;0.14)	>0.999
Glucocorticosteroids (latest 12 months)	9 (52.9)	81 (37.7)	0.08 (0.01;0.22)	0.325
Anti-TNF (latest 12 months)	0 (0.0)	36 (16.7)	0.12 (0.09;0.16)	0.082
Operation (latest 12 months)	0 (0.0)	20 (9.3)	0.09 (0.06;0.12)	0.374
Fracture	8 (47.1)	10 (4.7)	0.41 (0.18;0.64)	<0.001
Arthralgia	9 (52.9)	109 (50.7)	0.01 (0.00;0.15)	>0.999
Bone disease				
None	0 (0.0)	137 (63.7)	-	<0.001
Osteopenia	0 (0.0)	78 (36.3)		
Osteoporosis	17 (100.0)	0 (0.0)		
CTX, mg/mL	0.49 (0.49;0.57)	0.43 (0.28;0.70)	0.06 (0.01;0.22)	0.045
Osteocalcin, ng/mL	12.70 (10.90;19.20)	22.00 (13.50;31.10)	−9.30 (−12.10;−1.20)	0.007
PTH, pg/mL	45.70 (28.90;65.30)	36.20 (26.00;47.60)	9.50 (−0.90;16.40)	0.115
Vit. D, ng/mL	14.60 (8.80;15.70)	16.30 (9.10;21.20)	−1.70 (−5.80;1.30)	0.199

Data are presented as *n* (% of group), mean ± standard deviation, or median (1st quartile; 3rd quartile), depending on the type of variable and the normality of distribution in the case of numeric variables. MD—mean or median difference (osteoporosis vs. no osteoporosis and osteopenia vs. no osteopenia) for numeric variables, depending on the normality of distribution. Cramer’s V available in the case of categorical variables. Groups compared with independent Student’s *t*-test, Welch’s independent t-test, and the Mann–Whitney U test for numeric variables, as appropriate, and Pearson’s Chi-square test and Fisher’s exact test for categorical variables, as appropriate.

**Table 3:** Logistic regression model for osteoporosis in patients with IBD.

Variable	Patients with IBD|Univariate Model					Patients with IBD|Multivariate Model				
	Coeff	SE	OR	95% CI	*p*	Coeff	SE	OR	95% CI	*p*
Age, years	−0.02	0.02	0.98	0.94–1.02	0.406	−0.28	0.12	0.76	0.56–0.92	0.021
Gender, male (vs female)	1.74	0.65	5.68	1.79–25.14	0.008	8.69	3.94	5937.15	19.70–4.40 × 10^8^	0.028
BMI	−0.02	0.05	0.98	0.88–1.08	0.768	-	-	-	-	-
Crohn’s disease (vs ulcerative colitis)	−1.67	0.55	0.19	0.06–0.53	0.003	−7.35	2.73	0.00	0.00–0.05	0.007
Localization CD, small intestine	−0.02	0.55	0.98	0.30–2.77	0.975	-	-	-	-	-
Localization CD, large intestine	0.22	0.56	1.24	0.38–3.52	0.696	-	-	-	-	-
Localization UC, pancolitis	2.29	0.56	9.89	3.47–32.50	<0.001	-	-	-	-	-
Smoking	-	-	-	-	-	11.17	6842.21	71,174.32	0.00–1.41 × 10^105^	0.999
Rarely (vs every day)	1.16	0.57	3.2	1.10–10.66	0.040	5.93	2.62	374.85	7.74–339,510.40	0.023
Gymnastics	0.57	0.56	1.77	0.54–5.06	0.309	-	-	-	-	-
Other physical activity type	1.69	0.55	5.42	1.93–17.60	0.002	4.93	2.32	138.50	3.03–34,011.20	0.033
Calcium in diet	0.48	0.56	1.62	0.49–4.62	0.388	-	-	-	-	-
Supplementation of vit. D and Ca	−0.08	0.55	0.92	0.28–2.59	0.881	-	-	-	-	-
Glucocorticosteroids (last 12 months)	0.62	0.51	1.86	0.69–5.14	0.219	-	-	-	-	-
Fracture	2.90	0.58	18.22	5.79–58.70	<0.001	0.86	1.27	2.37	0.14–35.68	0.496
Arthralgia	0.09	0.50	1.09	0.40–3.02	0.859	-	-	-	-	-
CTX, ng/mL	0.76	0.78	2.14	0.42–9.40	0.331	-	-	-	-	-
Osteocalcin, ng/mL	−0.07	0.03	0.94	0.88–0.98	0.018	-	-	-	-	-
PTH, pg/mL	0.02	0.01	1.02	0.99–1.04	0.211	-	-	-	-	-
Vit. D, ng/mL	−0.06	0.04	0.94	0.87–1.00	0.100	-	-	-	-	-

Coeff—coefficient, SE—standard error, OR—odds ratio, CI—confidence interval.

**Table 4:** Patients with osteoporosis in the IBD and the control group.

Variable	With IBD	Control Group	MD/Cramer V (95% CI)	*p*
*N*	17 (100.0)	15 (100.0)	-	-
Age, years	39.29 ± 5.03	46.87 ± 12.96	−7.57 (−14.51;−0.64)	0.033
Gender				
Female	3 (17.6)	10 (66.7)	0.50 (0.14;0.81)	0.014
Male	14 (82.4)	5 (33.3)		
BMI	23.60 (15.51;23.60)	23.60 (18.00;29.00)	0.00 (−5.49;5.49)	0.801
Smoking	0 (0.0)	2 (13.3)	0.27	0.212
Physical activity				
Every day	5 (29.4)	0 (0.0)	0.53	0.015
Once a week	0 (0.0)	3 (20.0)		
Rarely	12 (70.6)	11 (73.3)		
Not physically active	0 (0.0)	1 (6.7)		
Calcium in diet	5 (29.4)	4 (26.7)	0.03 (0.00;0.38)	>0.999
Supplementation of vit. D and Ca	5 (29.4)	2 (13.3)	0.19 (0.01;0.49)	0.402
Fracture	8 (47.1)	8 (53.3)	0.06 (0.00;0.44)	>0.999
Arthralgia	9 (52.9)	2 (13.3)	0.42 (0.11;0.70)	0.048
T-score femoral neck	−1.31 ± 1.74	−2.30 (−2.60;−1.20)	0.00 (−0.40;0.40)	0.922
Z-score femoral neck	−2.10 (−2.30;−1.00)	−2.10 (−-2.20;−1.00)	0.00 (−0.20;0.20)	0.953
T-score lumbar spine	−2.90 (−4.90;−1.50)	−2.50 (−3.90;−2.00)	−0.40 (−1.00;1.00)	0.907
Z-score lumbar spine	−3.90 (−4.90;−1.10)	−2.40 (−4.40;−1.60)	−1.50 (−1.30;1.00)	0.861
CTX, mg/mL	0.49 (0.49;0.57)	0.49 (0.49;0.57)	0.00 (0.00;0.09)	0.742
Osteocalcin, ng/mL	12.70 (10.90;19.20)	12.70 (10.90;19.55)	0.00 (−4.50;4.50)	0.954
PTH, pg/mL	45.70 (28.90;65.30)	45.70 (27.95;55.50)	0.00 (−11.90;16.80)	0.684
Vit. D, ng/mL	14.60 (8.80;15.70)	14.60 (8.80;15.70)	0.00 (−5.60;1.70)	0.848

Data are presented as n (% of group), mean ± standard deviation, or median (1st quartile; 3rd quartile), depending on the type of variable and the normality of distribution in the case of numeric variables. MD—mean or median difference (IBD group vs. control group) for numeric variables, depending on the normality of distribution. Cramer’s V available in the case of categorical variables. Groups compared with independent Student’s *t*-test and Mann–Whitney U test for numeric variables, as appropriate, and Pearson’s Chi-square test and Fisher’s exact test for categorical variables, as appropriate.

## Data Availability

Data supporting reported results are available upon request.
